# Alumina and Zirconia-Reinforced Polyamide PA-12 Composites for Biomedical Additive Manufacturing

**DOI:** 10.3390/ma14206201

**Published:** 2021-10-19

**Authors:** Damian S. Nakonieczny, Frank Kern, Lukas Dufner, Magdalena Antonowicz, Krzysztof Matus

**Affiliations:** 1 Institute for Manufacturing Technologies of Ceramic Components and Composites, University of Stuttgart, 70569 Stuttgart, Germany; Frank.Kern@ifkb.uni-stuttgart.de (F.K.); Lukas.Dufner@ifkb.uni-stuttgart.de (L.D.); 2 Department of Biomaterials and Medical Devices Engineering, Faculty of Biomedical Engineering, Silesian University of Technology, Roosevelta 40 St., 41-800 Zabrze, Poland; Magdalena.Antonowicz@polsl.pl; 3 Department of Mechanical Engineering, Silesian University of Technology, Akademicka 2A, 44-100 Gliwice, Poland; Krzysztof.Matus@polsl.pl

**Keywords:** polymer–ceramic composites, polyamide PA-12, surface modification, zirconia, alumina, FDM printing, soaking test

## Abstract

This work aimed to prepare a composite with a polyamide (PA) matrix and surface-modified ZrO_2_ or Al_2_O_3_ to be used as ceramic fillers (CFs). Those composites contained 30 wt.% ceramic powder to 70 wt.% polymer. Possible applications for this type of composite include bioengineering applications especially in the fields of dental prosthetics and orthopaedics. The ceramic fillers were subjected to chemical surface modification with Piranha Solution and suspension in 10 M sodium hydroxide and Si_3_N_4_ to achieve the highest possible surface development and to introduce additional functional groups. This was to improve the bonding between the CFs and the polymer matrix. Both CFs were examined for particle size distribution (PSD), functional groups (FTIR), chemical composition (XPS), phase composition (XRD), and morphology and chemical composition (SEM/EDS). Filaments were created from the powders prepared in this way and were then used for 3D FDM printing. Samples were subjected to mechanical tests (tensility, hardness) and soaking tests in a high-pressure autoclave in artificial saliva for 14, 21, and 29 days.

## 1. Introduction

Polymer–ceramic composites (PCCs) have become increasingly prominent and found uses in biomedical applications with a particular emphasis in the field of dental prosthetics and orthopaedics. Much attention has focused on composite materials for osteosynthesis, treatment of bone defects, and prosthetic restorations such as crowns, bridges, and whole implant screws [1,2,3,4]. The popularity of PCCs stems from the fact they more closely resemble the human body and human skeletal system than metals or metal alloys. Furthermore, PCCs do not suffer from problems such as corrosion. Biomechanical mismatches between tissue and implant can lead to implant loss and tissue damage [5,6]. This issue mainly lies in the fact that the mechanical properties of human bones are individually variable. For the femoral cortical bone, the following mechanical values/ranges have been observed [7]: (I) in the longitudinal direction, an elastic modulus of 17,900 ± 3900 MPa; (II) in the transverse direction, an elastic modulus of 10,100 ± 2400 MPa. Implant materials have reported the following data: (I) for SS 316 L steel, an average of 202 GPa; (II) for Titanium alloy Ti6Al4V, an average of 110 GPa [8,9]; (III) for ZrO_2_ 3-YSZ, 210÷240 GPa; and (IV) for Al_2_O_3_, 380 GPa. For alternative materials on the PEEK matrix, depending on the volume share and filler chemistry, ranges from 5 GPa (TiO_2_-PEEK) to 6 GPa (lithium ceramic-SiO_2_-PEEK) to approximately 36 GPa (CF-PEEK) have been observed [10,11]. In orthopaedics, the corrosion and diffusion of metallic ions in the body have received considerable attention. Reports of adverse phenomena related to the diffusion of metallic ions have even been reported for titanium alloys, which have so far been considered as bio-inert [12]. Tardelli noted that Ti-6Al-4 V caused the release of ions and even intoxication, which caused the patient sensorimotor axonal neuropathy and bilateral sensorineural hearing loss. These health changes were due to high concentrations of vanadium ions in blood and urine samples from a hip prosthesis [12]. Other reports mentioned the role of protein adsorption in the corrosion of metallic implants [13]. With implants made of metals, especially those used in orthopaedics, tribological wear that resulted in accelerated corrosion and metallic ion release into human tissues has received significant attention [14,15]. Wang pointed out that long-term wear of Co-Cr-Mo hip joint prostheses and corrosion through body fluids, and the resulting formation of tribological films, wear particles, metal ions, and corrosion products, were the primary reasons for the degradation of joint prostheses [14]. Other studies have consistently pointed out the imperfections of metal implants and the need to modify their surfaces, especially those involving carbon compounds (graphene, graphene oxide) and silane layers to reduce the negative impact on the human body [16,17].

PCCs are used in prosthetics for several reasons such as the possibility of obtaining very good aesthetic effects for prosthetic restorations and the approximation of mechanical properties, including abrasion to human enamel [18,19]. Composite materials of this class are dedicated to both CAD/CAM and additive manufacturing systems [19,20]. The use of PCCs avoids the disadvantages of all-ceramic materials such as ZrO_2_ and Al_2_O_3_, which, due to biomechanical mismatches, may excessively abrade the enamel, or for certain diseases like bruxism, more readily undergo decomposition in the patient’s mouth [21,22,23,24]. Ning pointed out that PCCs, as compared to full ceramic materials, combine the advantages of polymers and ceramics and offer an interesting alternative due to their great aesthetics and outstanding machinability [19]. Dental clinics currently utilize PCC materials extensively, though they suffer from low mechanical properties and poor wear resistance [19]. Therefore, they still represent an area for development. Krishnakumar and Senthilvelan mention new PCCs that are suitable for the manufacture of fixed prostheses, i.e., bridges and crowns, including full dental arches, due to their appropriate mechanical properties [18]. Those suitable PCCs include CF/PMMA, UHMWPE/PMMA, and GF/PMMA [18].

The main technological problems of PCCs, regardless of the processing method (injection molding or additive manufacturing), occur from the improper adhesion between the polymer matrix and the ceramic and homogeneous filler dispersions in the matrix [25,26]. Obtaining a homogeneous filler dispersion in the matrix is necessary to obtain a smooth composite surface, flexural and fatigue strength, controlled shrinkage, and crack resistance during polymerization, and to ensure the same physical and chemical properties throughout the material. [27,28]. This phenomenon applies to all filler groups (organic and inorganic), shapes (nanorods, whiskers, and spheres), and sizes (nanometric to micrometric) [29,30]. A solution to the problem of heterogeneous filler dispersion in a polymer matrix is to modify the filler surface [31,32,33,34]. Some active groups (OH–, COOH–, NH2–) in the modified fillers, which can react with the polymer matrix physically and/or chemically, undergo additional interactions and enhance the interfacial adhesion between the matrix and filler. Modification of the filler surface ensures good wettability throughout the polymer matrix and yields a homogeneous dispersion [32,33]. Non-wetting between the polymer matrix and the filler is the main reason why fillers tend to form coatings on the preform surface to give a non-uniform distribution in the reinforcement because it increases the friction resistance between the filler and matrix [32]. Therefore, this often requires surface modification before joining them in a composite. Silanization is a type of modification often used in biomedical grade ceramics. This allows the introduction of groups involved in crosslinking onto the filler surface [33]. An example of such groups involves methacrylic moieties; these are introduced using 3-methacryloxypropyltrimethoxysilane (MPS) as the modifier, which bonds with montmorillonite via its hydroxyl and siloxane groups [33]. The available literature provides only sporadic information on compounds used for silanization (3-aminopropyltriethoxysilane (APTES), acetone-imine propyl trimethoxysilane (AIPTMS), 3-(methacryloyloxy)propyltrimethoxysilane (MPS), hexamethyldisilazane, mercaptopropyltrimethoxysilane (MPTMS), and Si_3_N_4_) [32,33,35,36,37,38].

The main objective of this study was to develop a new type of PCC, dedicated to medical applications processed by fused deposition modelling (FDM). PA (VESTAMID PA12, Evonik) was selected as the polymer matrix, while alumina (Sumitomo, Sumicorundum AA-18) and zirconia (ZrO-T6) were used as ceramic fillers (CFs); they were modified with Si_3_N_4_ (Höganäs grade B7) to improve adhesion to the polymer and solve the filler dispersion problem in the composite. First, both CFs were subjected to a two-step surface modification—etching with a hot Piranha Solution followed by Si_3_N_4_ surface modification in 10 M NaOH. The powders were then neutralized, dried and sieved. Powders prepared this way were mixed with PA and a filament for a 3D printer by FDM was prepared from them. From the filament, samples were prepared for mechanical and soaking tests in simulation body fluid—SBF (artificial saliva) in a high-pressure autoclave. The chemical and phase composition, surface morphology, and grain size of raw and modified fillers were characterized. The composite was tested for hardness and tensile strength, and testing occurred before and after soaking in an autoclave.

## 2. Materials and Methods

### 2.1. Sample Preparation

The ceramic powders α-alumina (Sumitomo, Sumicorundum AA-18) and unstabilized zirconia (ZRO-T6 IMERYS) were subjected to a two-stage surface modification. First, both fillers were etched in fresh, hot Piranha Solution, which was prepared using H_2_SO_4_ (CAS:7664-93-9, Carl ROTH) and 30% H_2_O_2_ (CAS:7722-84-1, MERCK) in a 3:1 volume ratio. The acid was stirred in a beaker with a flat bottom on a magnetic stirrer (time, 10 min; speed, 350 rpm) (CHEMLAND, Starogard Szczeciński, Poland). After 10 min, CFs were added and treated with the Piranha Solution for 30 min (speed, 350 rpm). After 30 min, the Piranha Solution was decanted into a separate beaker. To remove residual acid, the powders were filtered (4 × 1000 cm^3^ wash) with deionized water under reduced pressure using a water pump followed by washing with 2-propanol (CAS: 67-63-0, Carl ROTH). A 10M NaOH solution (CAS: 1310-73-2, Carl ROTH) was prepared into which Si_3_N_4_ (Höganäs, grade B7) was added. The mass ratio of NaOH:Si_3_N_4_ was 10:1 (100:10 g). After stirring for 1 h (speed, 350 rpm), the ceramic powders were added. After stirring for 2 h, the solution was decanted and the powders were neutralized with 1 M citric acid (CAS: 77-92-9, Carl ROTH). The powders were then transferred to a thermostated ball mill (Heynau H-Treib) (Heynau, Landshut, Germany) that included 2-propanol and ZrO_2_ grinding balls. The wet grinding time of the powders was 1 h. After grinding, the powders were transferred to metal bowls and a forced-air dryer (BINDER, Tuttlingen, Germany) (drying time, 24 h; temperature, 60 °C). Once dried, the powders were transferred to a laboratory sifter (Retsch) and sieved through 400 µm mesh (Retsch). Powders prepared this way served as a filler for the preparation of a polymer-ceramic composite filament. Powder samples for physicochemical tests were labelled as follows, powders after etching Al_2_O_3__1 and ZrO_2__1 and powders after etching and chemical surface modification, Al_2_O_3__2 and ZrO_2__2.

### 2.2. Filament Preparation

To produce the polymer ceramic filaments, a twin-screw extruder for the compounding, and a single screw extruder for the filament preparation, were used. To avoid hydrolysis, the PA-12 (VESTAMID PA12, Evonik) granulate was pre-dried at 50 °C for 10 h, alumina and zirconia powders were dried at 150 °C for 10 h. The molecular weight of PA-12 ranged from 9100 to 16,600 gmol^−1^ [39]. The EBVP 25/44D extruder from O.M.C. SRL (Saronno, Italy) was used for compounding. The ceramic powder and the polymer granules were dosed gravimetrically with a mass ratio of 30% ceramic powder to 70% polymer (PA). The CFs content was determined experimentally based on trials. Contents greater than 30% caused print quality degradation and clogging of the FDM print head. A possible solution to this problem was to use an FDM printing modification with a movable piston that regulated the pressure at the head outlet [40]. The mass throughput was 4.2 kg/h at 100 rpm. The extruder temperature was selected above the melting temperature of PA and was 260 °C at the extruder exit. After compounding, the polymer ceramic strand was cooled using a water bath and then granulated. A single-screw extruder from DR. COLLIN GmbH (Ebersberg, Germany) was used for shaping. The mass throughput was 3 kg/h at 14 rpm. After extrusion, the polymer ceramic melt was pulled with a pull-off force, which depended on the crystallization degree of the carrier material. To set the pull-off force, filament diameters between 1.6 and 1.8 mm were ensured, recorded using a WIREMASTER and the ODAC 18 XY laser head from Zumbach (Orpund, Switzerland). The material used for comparison in batch 4 was a commercially available white 1.75 mm eco PLA filament from 3DJAKE (Niceshops GmbH, Paldau, Austria).

### 2.3. Filament Mechanical Testing Sample Preparation

Initially, two geometric samples were printed using a PA-ZrO_2_ filament and an extruder temperature between 205 and 260 °C with stepwise temperature increases of 5 °C to assess the processable temperature range needed to obtain a qualitative satisfactory surface quality and interlayer bonding. Using the same material, 30 samples, type 1BA, of the EN ISO 527-2:2012 norm 4 mm thick were printed vertically at temperatures between 230 and 255 °C with stepwise temperature increases of 5 °C (batch 1), to determine the optimal temperature for interlayer binding. The infill density of these tensile specimens was set to 90% to reduce instabilities during printing of the upper layers caused by the high sample aspect ratio. Both batches were processed using a commercial Ender 3 Pro printer from Creality(Creality, Shenzhen, China), which was converted for printing ceramics by replacing the extruder with the Micro Swiss Direct Drive Extruder (Creality, Shenzhen, China) to guarantee a stable feed rate and print ceramic filaments with limited abrasion from the drive gears. To improve adhesion, the print bed was replaced with an Ultrabase glass print bed from Anycubic (Anycubic, Shenzhen, China).

During the second step, batches 2 and 3 were printed using the optimized interlayer binding temperature (250 °C cf.), and for comparison, batch 4 was printed using the conventional processing temperature for PA and the same processing parameters. Batches 2, 3, and 4 were printed on a i3 MK3 from Prusa (Prusa, Praha, Czech Republic), Research with a flexible steel sheet print bed. After printing, the samples were maintained at 23 °C and with a relative humidity of 20%. Table 1 shows the process parameters for the tensile specimens.

The hardness samples were printed using the same parameters as batches 2 and 3. The samples were printed and cold mounted in resin, both in the z (vertical) and x (perpendicular to the raster orientation) directions and then ground, dried, and maintained at 23 °C and with a relative humidity of 20%. Mechanical characterization included Vickers hardness measurements (HV10, 98.1 N applied for 10 s, five samples, Bareiss, Oberdischingen Germany). 

### 2.4. Soaking Test Samples Preparation

**Figure 1:**
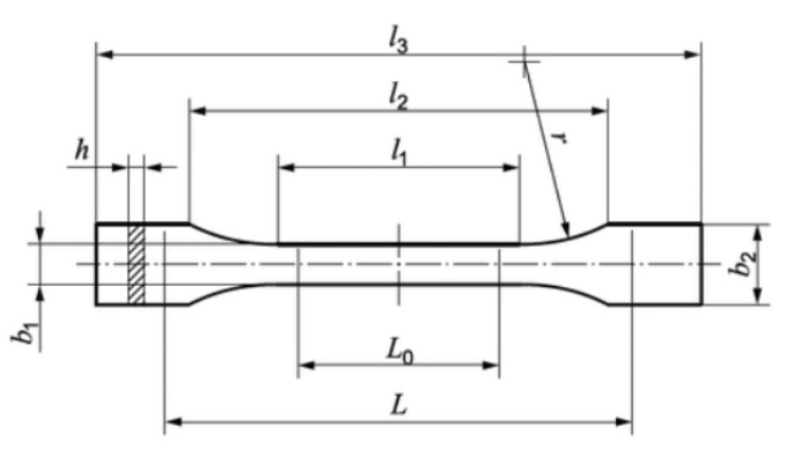
Sample type 1BA for strength test [41].

In order to assess the effect of exposure to artificial saliva on the mechanical properties of samples made of PA12-ZrO_2_ and PA12-Al_2_O_3_ filament, a series of samples were prepared by FDM printing on another 3D printer (Double P255 by 3D Gence, Gliwice, Poland). In accordance with the recommendations of ISO 527:2012 [A], type 1BA samples were prepared, which are preferred for machined specimens. The characteristic dimensions of the samples are shown in Figure 1 and Table 2. The 3D models were modeled in SolidWorks 2020 software. In order to read the file by the printer, the file was saved in the STL format, and the appropriate printing parameters were selected using 3D Gence Slicer 4.0. software.

In the first stage, geometric samples for both materials were printed at extruder temperatures between 200 °C and 220 °C to verify the quality of the surface and interlayer bonds, and then to select the appropriate temperature. A group of 30 samples for each material was printed at 210 °C without cooling, due to the good print quality. The printing parameters are shown in the Table 3. The printed samples were stored at 23 °C and 20% relative humidity.

#### Soaking Test

Samples from both composites were kept sealed in high-pressure autoclaves (Carl ROTH, Model-1) (Carl Roth, Karlsruhe Germany) filled with artificial saliva (chemical composition, Table 4, according to EN ISO 10993-15:2000). Autoclaves ware placed in a forced-air dryer (Binder, Tuttlingen, Germany) at 37 °C for 14, 21, and 29 days. After the exposure time in artificial saliva, the samples were subjected to mechanical tests.

### 2.5. Methods

#### 2.5.1. X-ray Diffraction (XRD)

The powder compositions before and after etching were determined by XRD (X’Pert MPD, PANalytical Germany) in the 2θ-range between 20 and 70° (X’Pert MPD, Ge-monochromator, accelerator detector).

#### 2.5.2. Particle Size Distribution (PSD)

PSD was measured using a laser granulometer Mastersizer 3000 with the Hydro LV wet sample dispersion unit (Malvern Panalytical, Malvern, United Kingdom). The modified ceramic powders were dispersed in distilled water in a 50/50 wt.% suspension, adding 0.5 wt.% of the overall weight of dispersant DOLAPIX CE64 (Zschimmer & Schwarz, Lahnstein, Germany). The mixture was added into the water-based measuring cell until the obscuration level reached approximately 10%. The assumed refractive indexes for particle size calculations according to the Mie model were 1.76 for Al_2_O_3_ and 2.165 for ZrO_2_.

#### 2.5.3. Scanning Electron Microscopy with Energy-Dispersive X-ray Spectroscopy (SEM/EDS)

##### Powders

Microstructure observations of the powders were made using a ZEISS SUPRA 35 microscope (Zeiss, Jena, Germany) and secondary electron (SE) and backscattered electron detection at an acceleration voltage of 10 kV and a maximum magnification of 50,000×. For EDS, an UltraDry EDS Detector (Thermo Scientific™ Pathfinder™ X-ray Microanalysis Software, Waltham, MA, USA) determined the chemical composition of the analysed samples.

##### Filaments

The filament cross-sections, tensile specimen cross-sections, and fracture surfaces were prepared using a Leica EM SCD005 sputter coater (Leica Microsysteme GmbH, Wetzlar, Germany) for 30 s at 20 mA and observed using a JCM-5000 SEM (JEOL Ltd., Tokyo, Japan) at an acceleration voltage of 10 kV at different magnifications.

#### 2.5.4. Fourier Transform Infrared Spectroscopy (FTIR)

FTIR was used to identify characteristic functional groups. Spectra were obtained using a Shimadzu IR Tracer-100 FTIR Spectrophotometer (Shimadzu, Kioto, Japan) (Michelson interferometer; beam splitter, KBr germanium coated; light source, high-energy ceramics; detector, DLATGS detector) using a multi-reflection ATR attachment equipped with a diamond prism. To conduct the correct analysis, the device was calibrated with a closed ATR attachment that recorded the background image. Test samples were then placed on the diamond and pressed against the prism with a dynamometric screw using the same force for each sample. Transmission spectra were recorded on a multi-reflection device to analyse and interpret the characteristic bands of each sample (from 4000–400 cm^−1^). Analyses were performed automatically using dedicated LabSolution IR software provided by the spectrometer manufacturer. To minimize the error, 100 counts were performed with a resolution of 4 cm^−1^ for each analysis.

#### 2.5.5. X-ray Photoelectron Spectroscopy (XPS)

XPS investigations were carried out in a multi-chamber ultra-high vacuum experimental setup (base pressure 2 × 10^−8^ Pa) equipped with PREVAC EA15 hemispherical electron energy analyser (Prevac, Rogów, Poland) with a 2D-MCP detector. The samples were irradiated using an Al-Kα X-ray source (PREVAC dual-anode XR-40B source, energy 1486.60 eV). For survey spectra, the pass energy was set to 200 eV (with scanning step 0.9 eV). For specific spectra, the pass energy was set to 100 eV (with scanning step 0.05 eV). The binding energy (BE) scale of the analyser was calibrated to Au 4f7/2 (84.0 eV) [42]. Recorded data were fit utilizing CASA XPS^®^ embedded algorithms. For composition determination, the universal transmission function and relative sensitivity factors [43] were applied. The Shirley function was used for background subtraction. If not specified, the components were fit with a sum of Gaussian (70%) and Lorentzian (30%) lines. The full width at half maximum (FWHM) values for the peaks at the same binding energy region were allowed to vary within a narrow range.

#### 2.5.6. Filament Mechanical Testing

Tensile tests were conducted on a universal testing machine (Zwick Z100, Zwick GmbH & Co. KG, Ulm, Germany) at a crosshead speed of 0.5 mm/min and a preload of 2 N for Batch 1 and a crosshead speed of 0.5 mm/min and a preload of 15 N for Batches 2, 3, and 4 per the EN ISO 527-1:2019 norm. The strain was measured directly on the machine without the use of an extensometer. As mentioned above, the samples were conditioned, kept in a room at 23 °C with 20% relative humidity. Because of the irregular sample surfaces, the section was set as the theoretical cross-section defined in the CAD model (20 mm^2^). The ultimate tensile strength is calculated as follows:

$$\sigma_{m}=\frac{F_{max}}{A}$$

where, *σ_m_* is the ultimate tensile strength in Megapascals (MPa), *F_max_* is the maximum force measured during the tensile test in Newton (N), and *A* is the theoretical initial cross-section in square millimetres (mm^2^).

The nominal strain at the ultimate tensile strength is calculated using the following equation:

$$\varepsilon_{m}=\frac{L_{m}}{L}$$

where, *ε_m_* is the strain at which the strength *σ_m_* is reached as a percentage (%), *L_m_* is the length of the part between the grips at which the strength σ_m_ is reached, in millimetres (mm), and L is the initial length of the part of the specimen between the grips as defined by EN ISO 527-2:2012, in millimetres (mm).

Young’s modulus of the composite tensile specimens was defined with the regression of the stress/strain curve between 1% and 2% strain, deferring from the norm because of the curve offset during the initial load application.

$$E_{t}=\frac{d\sigma }{d\varepsilon }$$

where, *E_t_* is the tensile modulus in Gigapascals (GPa) and *dσ/dε* is the slope of the regression curve in Gigapascals (GPa).

Vickers hardness HV1 measurements (Bareiss Prüfgerätebau GmbH, Oberdischingen, Germany) were performed with a 1 kgf load applied for ten seconds. The resulting Vickers hardness represents the mean of six measurements with a measured length difference of the indentation diagonals below 5%.

#### 2.5.7. Soaking Samples Mechanical Testing

The static tensile test was conducted in accordance with the recommendations of the standard EN ISO 527:2012 with a tensile speed of 5 mm/min, using the MTS Criterion Model 45 machine (MTS, Eden Prairie, MN, USA) with a 10 kN force sensor and MTS TestSuite software. The separation between the grippers was 54 mm. Based on the test, the maximum breaking force Fmax (N), the Young’s modulus E (MPa), elongation at break A (%), and tensile strength Rm (MPa) were determined. A static tensile test was performed on PA12 + ZrO_2_ and PA12 + Al_2_O_3_ samples both before and after soaking in artificial saliva for 14, 21, and 29 days. Five studies were performed for each type of sample.

Measurements of microhardness for PA12 + ZrO_2_ and PA12 + Al_2_O_3_ in initial state and soaking samples were carried out using the instrumental method (Oliver and Pharr), which is a measure of the resistance of a material to permanent deformation or damage, is defined as the quotient of the maximum applied loading force and the projected area of the contact area between the indenter and the test sample [44]. The tests were carried out using the open platform equipped with a Micro-Combi-Tester by CSM Instruments (Micro-Combi-Tester, CSM instruments a company of Anton Paar, Peseux, Switzerland) using a Vickers indenter. The micromechanical properties were determined based on material deformation as a result of indentation of the sample with Vickers indenter to which a 100 mN maximal load was applied. The value of the loading force and the penetration depth of the indenter blade were recorded continuously during the entire cycle (loading and unloading). Loading and unloading rate was 200 mN/min, a hold time of the sample at maximum load—5 s. The value of the indenter load was the result. The microhardness result was the average of 10 measurements measured longitudinally and transversely across the entire surface area of the PA12 + ZrO_2_ and PA12 + Al_2_O_3_ samples in initial state and after exposure in artificial saliva. The values of indentation hardness, HIT, and Vickers hardness, HVIT, were determined.

## 3. Results and Discussion

### 3.1. Powder Analyses

**Figure 2:**
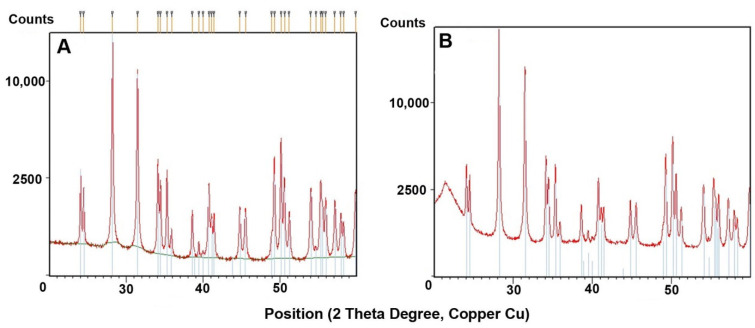
XRD patterns: (**A**) trace of as-received zirconia powder, (**B**) trace of 3D-printed zirconia composite.

**Figure 3:**
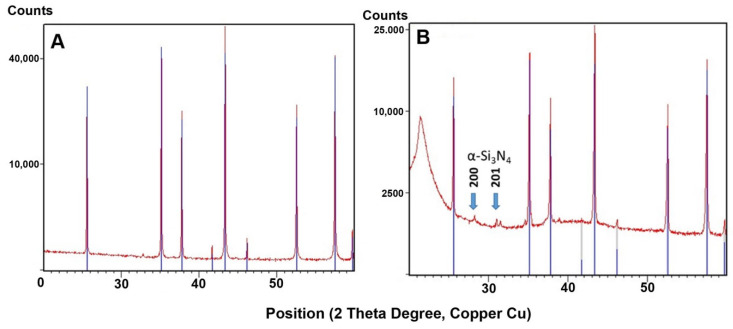
XRD patterns: (**A**) trace of as-received alumina powder, (**B**) trace of 3D-printed alumina composite.

Figure 2A,B shows the XRD traces of the as-received and 3D-printed zirconia composites. Both powders showed identical patterns corresponding to monoclinic zirconia JCPDS card no. 37-1484 (inserted lines). The XRD patterns of the crystalline phase were identical, though some amorphous polymer phase (the polymer) was visible at 22° 2θ. The minority fraction of the added silicon nitride was invisible due to the high intensity of the zirconia peaks. Figure 3A,B shows the XRD traces of the as-received alumina powder and 3D-printed alumina composite. The large peaks were identical and corresponded to α-alumina (JCPDS card Nr. 83-2080). The alumina was, therefore, not visibly modified by the etching, compounding, or printing process. For the 3D-printed material consisting of an alumina mixture with silicon nitride as a minority phase, small peaks from 25–35° (indicated by blue arrows) were attributed to α-Si_3_N_4_ (JCPDS card Nr. 41-0360). The largest reflex of silicon nitride (210) at 36° coincided with the 104 reflexes of alumina.

**Figure 4:**
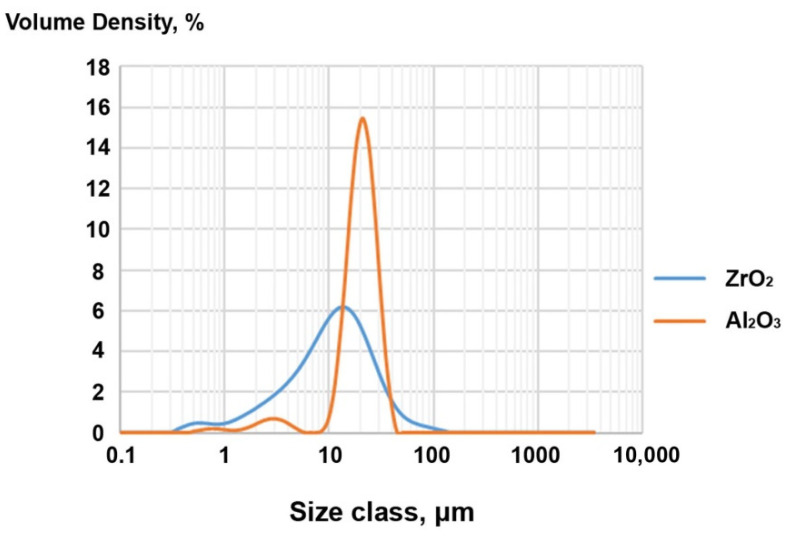
Particle size distribution of the modified ZrO_2_ and Al_2_O_3_ powders.

Figure 4 and Table 5 illustrate the particle size distribution, and those results reflect the mean of five measurements. The ZrO_2_ powder has a larger particle size distribution with most particles in the 1–10 μm range, as seen in Figure 4 below.

**Figure 5:**
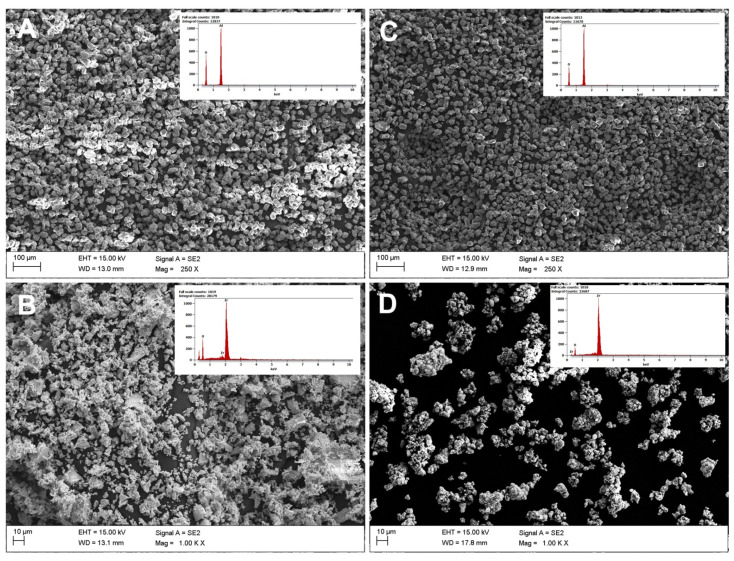
SEM/EDS micrographs of the powders: (**A**) raw alumina, (**C**) alumina after etching in Piranha Solution; (**B**) raw zirconia, (**D**) zirconia after etching in Piranha Solution.

Powder observations for Al_2_O_3_ and ZrO_2_ were made for raw samples before etching and powders after Piranha Solution digestion before adding Si_3_N_4_. Based on powder observations before and after etching, the degree of agglomeration in the powders after etching increased. Regarding the chemical composition, etching resulted in no negative effects and both samples had the same chemical compositions before and after etching. After digestion, the powders noticeably clumped into agglomerates (Figure 5).

**Figure 6:**
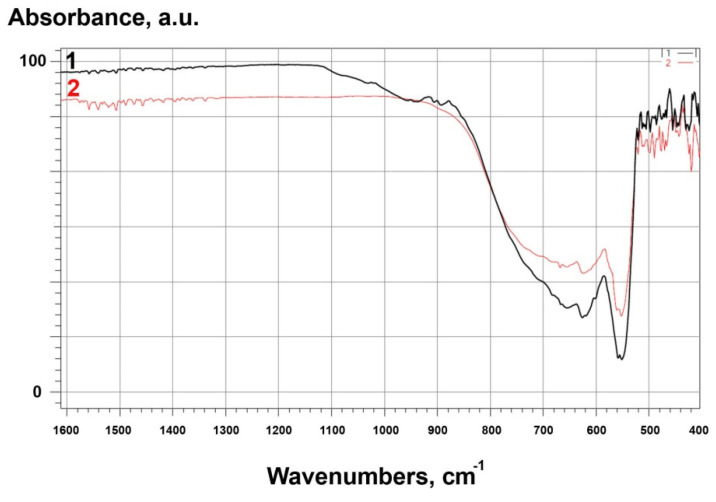
FTIR spectra for: 1-Al_2_O_3__2 (modified), 2-Al_2_O_3__1 (raw).

**Figure 7:**
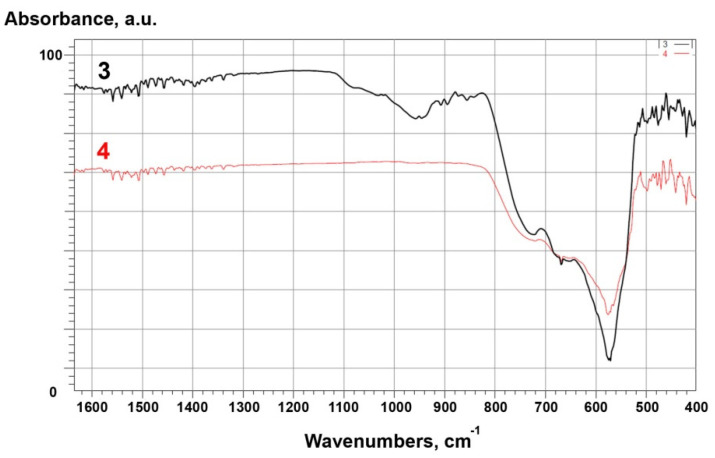
FTIR spectra for: 3 – ZrO_2__2 (modified), 4 – ZrO_2__1 (raw).

FTIR spectra for all samples are shown in Figure 6 and Figure 7. For raw alumina powders, the IR spectra corresponded to α-Al_2_O_3_ [45]. Bands at 1600 and 1500 cm^−1^ are attributed to H–O–H bonds. A band observed at 1080 cm^−1^ was the result of bending vibrations from hydroxyl groups bound to alumina. Absorption bands that appeared near 1030 cm^−1^ were related to vibrations, stretches, and deformations of O–H bonds present due to absorption and coordination of water in the samples [45]. Bands at lower wavenumbers 670, 640, 580, 550, and 460 cm^−^^1^ were attributed to a pseudoboehmite structure [46]. For modified alumina, bands at 880, 900, 920, 950, 1040, and 1075 cm^−1^ corresponded to β-Si_3_N_4_ [47].

For raw zirconia (ZrO_2__1), bands in the same regions were identified as for alumina. The band at 1080 cm^−1^ related to the bending vibrations of hydroxyl groups and bands at 1030 cm^−1^ related to the vibrations, stretches, and deformations of O–H bonds. The spectrum showed characteristic monoclinic zirconia bands at 720, 680 and 580 cm^−1^ [48]. Bands below 500 cm^−1^ were identified as metastable zirconia phases (tetragonal and cubic) [48]. On the other hand, for modified zirconia (ZrO_2__2), the presence of characteristic bands for β-Si_3_N_4_, previously identified for modified alumina, was confirmed [47].

**Figure 8:**
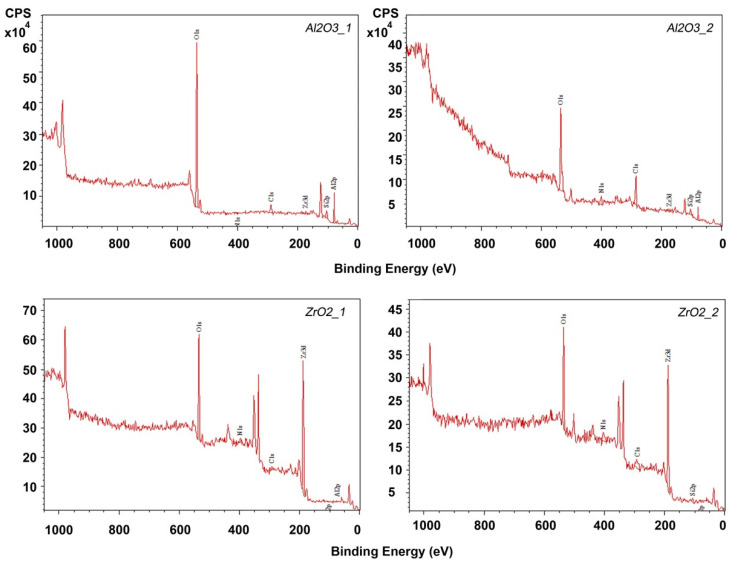
XPS survey spectra for powders etched in Piranha Solution and chemically modified.

XPS analysed the elemental composition of the modified ceramic powders and the exact chemical composition of the prepared powders was determined. Those results are presented in Table 6. Survey spectra are shown in Figure 8.

Chemical modification by etching had little effect on the grain size change. Based on SEM/EDS and PSD results, the following conclusions were drawn. The distribution for both powders was basically homogeneous. For zirconia, a smaller grain size but wider particle size distribution was observed (Figure 4). However, surface modification favoured powder agglomeration. This trend was observed for both alumina and zirconia (Figure 3). A significantly higher degree of aggregation was observed for zirconia (Figure 5) and can be explained by the occurrence of weak van der Waals forces that caused agglomeration of the ceramic powders [49,50,51]. That zirconia clumped into smaller agglomerates than alumina can be explained by the agglomeration potential, which is a function of spherical particle radii that increases with decreases in the individual ceramic powder particle sizes (Figure 4) [51]. At the same time, the agglomerate strengths increased only for very small particles. Interactions causing clumping into agglomerates resulted from etching and drying, evaporation of the dispersion medium, which affects the surface energy change, the fracture stress of the solid bridge, and the friction coefficient [51].

Based on FTIR, XPS, and XRD studies, the chemical and phase compositions and functional groups were determined. IR spectra (Figure 2, Figure 3, Figure 6, Figure 7 and Figure 8) confirmed the presence of –OH groups and Si_3_N_4_. Hydroxyl groups benefit ceramic surface modifications acting as polymer fillers because they prevent the formation of unfavourable surface energy differences between the hydrophilic ceramic and hydrophobic polymer matrix [52]. If such differences occurred, this would lead to agglomeration and poor dispersion of the ceramic filler particles within the polymer matrix. Hence, this chemical modification protects against the formation of voids and interfacial defects in PCCs [52]. Silanes perform a similar function, including organosilanes and nitrogen and silicon-based compounds like Si_3_N_4_.

XPS analysis confirmed the elemental composition of the fillers. For alumina and zirconia powders, the occurrence of the desired Si and N compounds from Si_3_N_4_ was confirmed. The occurrence of carbon in powders etched in the Piranha Solution and etched and modified with Si_3_N_4_ (Figure 8) was confirmed. Considering the used reactants, one possible source of contamination could be 2-propanol, used for washing powders after etching as the grinding medium in the ball mill. The full temperature decomposition of 2-propanol occurs between 129 and 189 °C [53]. Thermal processes conducted in this experiment took place at 60 °C, so it is possible that carbon impurities originated from residual 2-propanol. The XRD results correlated well with XPS analyses. Based on those diffractogram analyses, no significant phase composition changes were found, which could have been caused by etching, compounding, or printing (Figure 2 and Figure 3).

### 3.2. Filament Testing

#### 3.2.1. Filament and Sample Cross-Sections

**Figure 9:**
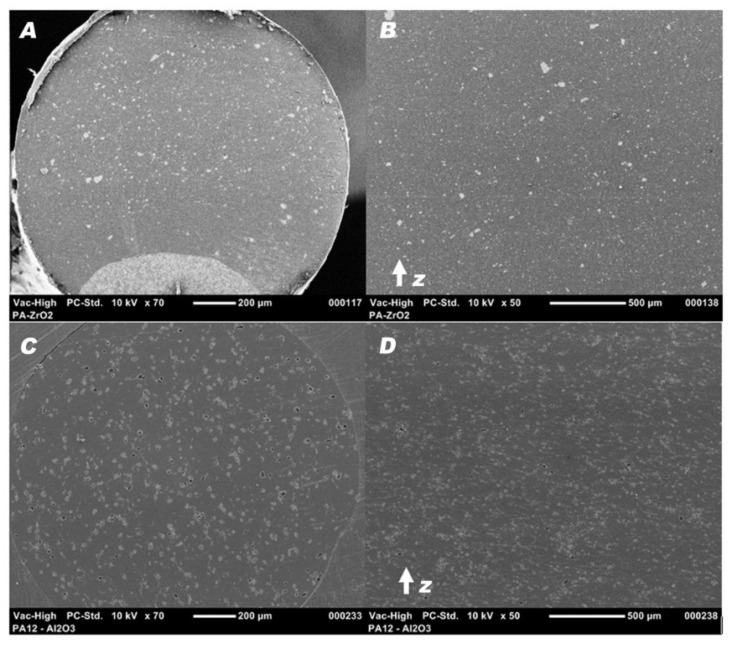
SEM micrographs of the filament at magnification ×70, (**A**) PA12-ZrO_2_, (**B**)PA12-Al_2_O_3_, and the cross-section of a tensile specimen at magnification ×50, (**C**) PA12-ZrO_2_, (**D**) PA12-Al_2_O_3_.

SEM micrographs of the ground filament and tensile specimen cross-sections showed a homogenous dispersion of the ceramic powders and no porosity. Furthermore, the tensile specimens exhibited no specific patterns, delaminations, or interlayer porosity in the *z*-axis build direction. The voids apparent on PA12-Al_2_O_3_ samples shown in Figure 9 were caused by the removal of larger particles during grinding.

#### 3.2.2. D Printing

##### Geometric Samples

**Figure 10:**
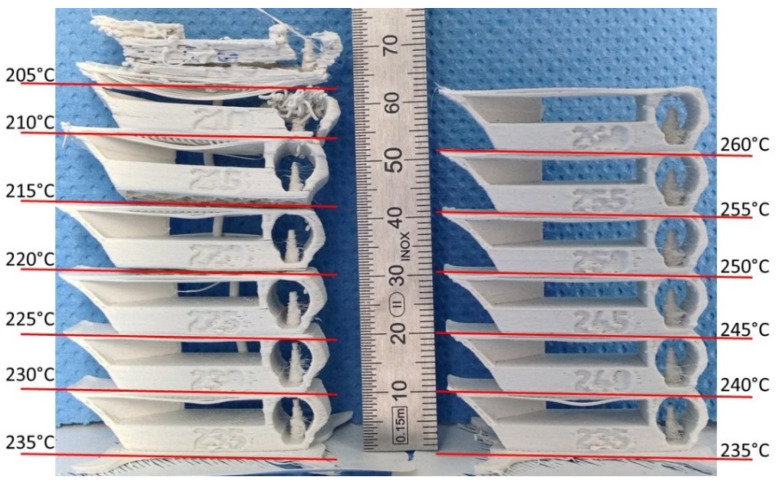
Parts printed at different temperatures showing the temperature range for the PA12-ZrO_2_ Fused Filament Fabrication.

As shown in Figure 10, for the PA12-ZrO_2_ composite, the printability of the highly filled PA12 filament was good at temperatures between 225 and 255 °C. It showed signs of delamination below 225 °C and poor surface and detail finish above 255 °C.

#### 3.2.3. Filament Mechanical Testing

##### Ultimate Tensile Strength, Elongation, and Young’s Modulus

**Figure 11:**
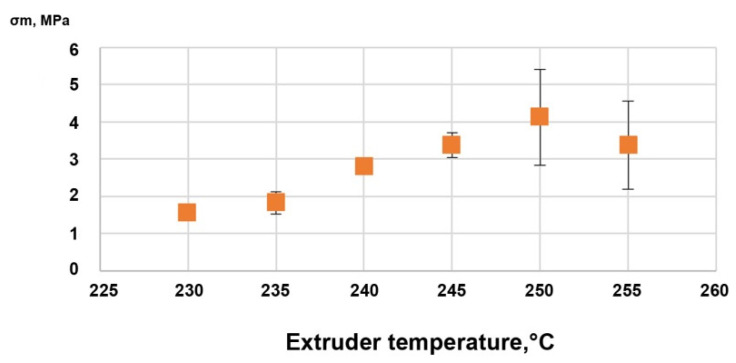
Ultimate tensile strength vs. printing temperature of the PA12-ZrO_2_ samples from Batch 1.

The ultimate tensile strengths of the vertically printed Batch 1 samples are displayed in Figure 11. The optimal temperature for interlayer bonding appeared to be 250 °C. This temperature was used to print Batch 2 samples.

**Figure 12:**
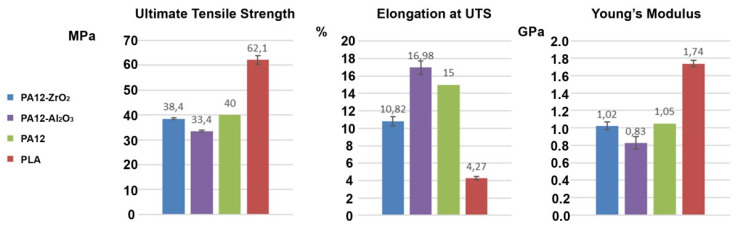
Ultimate tensile strength, elongation at ultimate tensile strength, and Young’s modulus of composite materials PA12-ZrO_2_, PA12-Al_2_O_3_, pure PA12, and PLA.

The specimen tensile test results from Batches 2, 3 (particulate composites), and 4 (PA) are shown in Figure 12 and compared to the mechanical properties given by the supplier of the PA12 used for the filament [48]. The ultimate tensile strengths of the composites were 38.4 ± 0.4 MPa for the PA12-ZrO_2_ and 33.4 ± 0.4 MPa for PA12-Al_2_O_3_, both of which were slightly below the PA12 UTS of 40 MPa. The strength strains of the composites were also lower than the pure material, which were 10.82 ± 0.54%, 16.98 ± 0.77%, and 15%, respectively. The same phenomenon occurred with the Young’s modulus; the PA12-ZrO_2_ composite had a Young’s modulus of 1.02 ± 0.04 GPa, and the modulus for composite PA12-Al_2_O_3_ was 0.83 ± 0.07 GPa, both of which were lower than the 1.05 GPa reported for pure PA12. For comparison, PLA specimens printed using the same parameters as Batches 2 and 3, but at temperatures adapted for PLA, showed a strength of 62.1 ± 1.7 MPa, a strain of 4.27 ± 0.17%, and a Young’s modulus of 1.74 ± 0.04 GPa.

**Figure 13:**
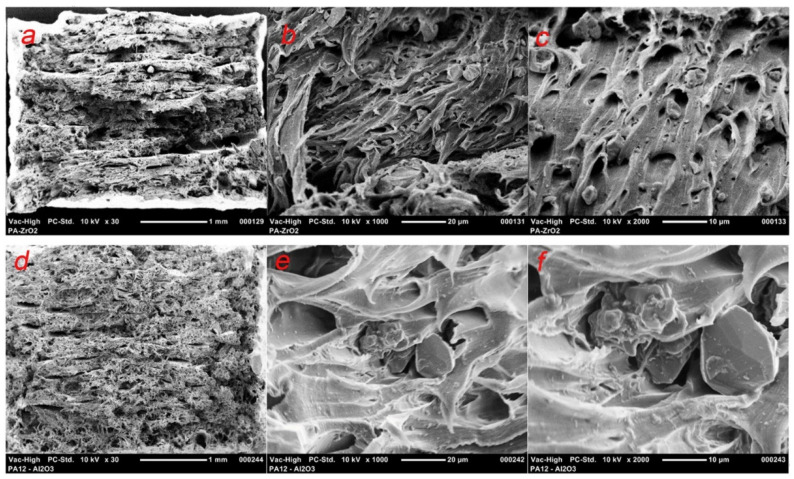
SEM micrographs of the fracture surface of PA12-ZrO_2_ at magnifications of ×30 (**a**), ×1000 (**b**), and ×2000 (**c**); PA12-Al_2_O_3_ at magnifications of ×30 (**d**), ×1000 (**e**), and ×2000 (**f**).

The fracture surface of the composites showed clear debinding between the ceramic particles and the polymer matrix (Figure 13). This is a common damage mechanism for these magnitudes and sizes of reinforcements with low interface strengths [50].

##### Vickers Hardness

**Figure 14:**
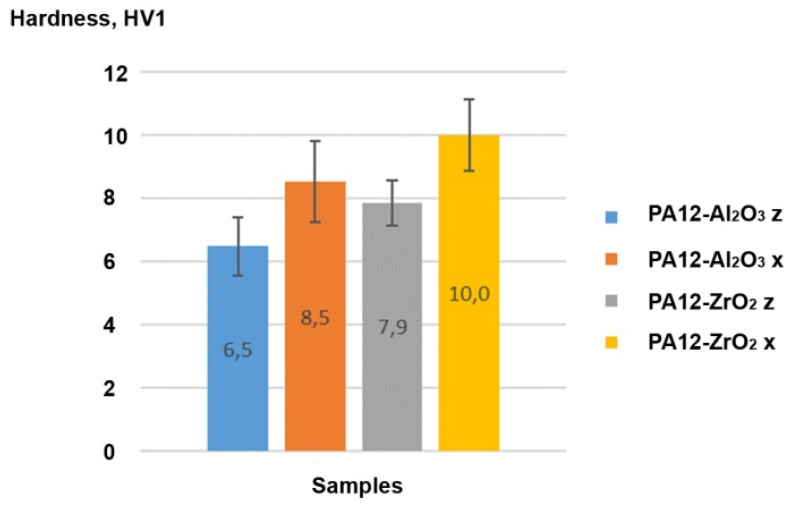
Vickers hardness of the PA12-Al_2_O_3_ and PA12-ZrO_2_ composites in z and x directions.

Vickers hardness values measured in the x-direction were higher than the z-direction, with 8.5 ± 1.3 HV1 and 6.5 ± 0.9 HV1 for Al_2_O_3_, and 10 ± 1.1 HV1 and 7.9 ± 0.7 HV1 for ZrO_2_, respectively (Figure 14). The diagonal length of the indentation was approximately 500 μm, the Dx90 of the powders was ~30 μm, and the powder was homogeneously spread across the section, so the measurements were representative for the composites’ hardness. On this basis, the hardness of the prepared composite was dependent on how the material was printed.

##### Soaking Test

**Figure 15:**
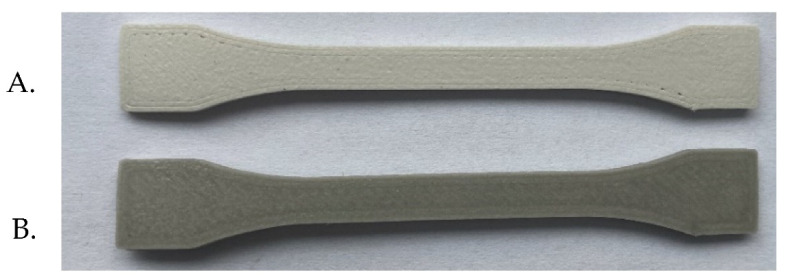
Samples for strength tests: (**A**). PA12-ZrO_2_, (**B**). PA12-Al_2_O_3_.

**Figure 16:**
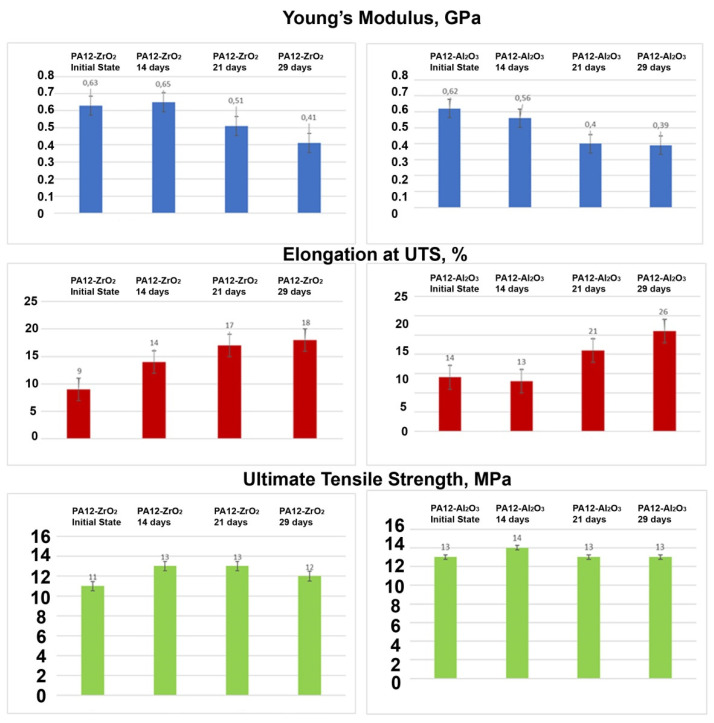
Young’s modulus, elongation at ultimate tensile strength, and ultimate tensile strength, of the composite materials PA12-ZrO_2_ and PA12-Al_2_O_3_.

The test samples are shown in Figure 15. The results of the tensile tests of the specimens PA12-ZrO_2_ and PA12-Al_2_O_3_ in initial state and after exposure in artificial saliva, after 14 days, 21 days, and 29 days are shown in Figure 16. The ultimate tensile strength of the composites in initial state were 11,5 MPa (±3.6 MPa) for the PA12-ZrO_2_ and 12,7 MPa (±2.3 MPa) for the PA12-Al_2_O_3_. After exposure in artificial saliva, the tensile strength increased with time for PA12-ZrO_2_, while for PA12-Al2O_3_ no significant changes were observed. However, the strain strength of both composites after soaking increased compared to the initial state. In contrast, Young’s modulus for both materials decreased with increased soaking time.

Test results of microhardness for all samples are presented in Table 7. Based on the data obtained it can be concluded that in the initial state PA12-Al_2_O_3_, (*HV_I_*_T_ = 14) is characterized by higher hardness in relation to the initial state of PA12-ZrO_2_ (*HV_IT_* = 17). Soaking PA12-ZrO_2_ samples in artificial saliva after 14 days resulted in a two-fold increase in hardness, while after 21 days it decreased and then after 29 days the hardness value increased. Instead, the PA12-Al_2_O_3_ samples showed a decrease in hardness after 14 days, a decrease after 21 days and an increase in hardness after 29 days. Measurements were taken in different areas of the samples and no differences were found across the entire area.

Based on SEM observations, there was uniform dispersion of filler grains in the polymer matrix (Figure 10). No agglomeration of fillers was observed. The shrinkage of the material during the printing caused a strong warping, which led to the loosening of the printed samples from the build plate, so suitable means were taken to avoid this effect. The influence of the filler share on the tensile strength was determined by mechanical testing. For zirconia filled PA the decrease in tensile strength was smaller in comparison to PA filled with alumina and the value was lower by only approximately 1.86 MPa in comparison to the tensile strength of bare PA as declared by the manufacturer. There is a limited number of papers of PA composites to which these results can be related [52,53,54]. The most interesting work was presented by Mohamad [54]. In their study they prepared a PA-12-ZrO_2_/β-TCP composite dedicated for craniofacial reconstruction [54]. For the 15-ZrO_2_-15 β-TCP-70PA (wt%) composite, they obtained a tensile strength of 24.25 MPa, with the bare PA made by EOS (PA2200, EOS, Germany) having a value of 33.98 MPa [54]. The result obtained in this study is higher, except that β-TCP is a porous ceramic dedicated to bone ingrowth and which may impair the mechanical properties of the composite. However, referring to the added percentages, the decrease in tensile strength of bare PA vs. zirconia filled PA in this study was obtained at 4% compared to 28.6% for Mohamad [54]. As far as the Young’s modulus values are concerned, the discrepancies between PA12-Al_2_O_3_ and the PA12-ZrO2 result from the mechanical properties of the fillers themselves and the bulk density of ceramic grains, for monoclinic zirconia powders the density is above 5.8 gcm^−3^, and for alumina 2.4 gcm^−3^ [55,56]. The smaller particle size results in a packed, dense composite structure and ultimately a higher Young’s modulus (Figure 5 and Figure 13). At the same time, it was noted that in comparing the obtained results of Young’s modulus values to those of Mohamed et al., it was found that the main factor influencing the value of the modulus of PA-12 composites was the mass ratio and the type of ceramic filler [54,57]. For solid ceramic fillers with higher density, Young’s modulus is higher with an obvious deterioration in elongation and ductility (Figure 13).

## 4. Conclusions

This was our initial effort to prepare polymer–ceramic composites for FDM 3D printing. The main aim of this study was to prepare polymer–ceramic composites consisting of PA-12 polyamide and surface-modified ZrO_2_ and Al_2_O_3_ powders. The purpose of the wet chemical Piranha Solution modification of the ceramic surface was to increase its surface area development and to introduce additional functional groups (like –OH) and compounds (like Si_3_N_4_) to improve the adhesion of the ceramic to the polymer and prevent unfavourable filler agglomeration in the polymer matrix and heterogeneous ceramic distribution. In this study, 30 wt.% zirconia or alumina were introduced into medical-grade PA-12 to create a composite with application potential in biomedical engineering, primarily dental prosthetics for fixed prostheses and temporary prosthodontics.

As a result of this work, we made the following key observations:

(1) chemical surface modification with Piranha Solution was a simple and effective method of ceramic surface modification and safer than using HF, though still time-consuming and requiring filtration and neutralization to achieve good results, leaving no impurities on the modified powders;
(2) the method of compacting and preparing the filament using a twin-screw extruder for the compounding and a single-screw extruder for the filament preparation produced composites with homogeneous filler dispersion in the polymer;
(3) after chemical modification, no negative changes in the phase composition of the ceramics were found; at the same time, –OH groups and uniformly-incorporated Si_3_N_4_ were observed;
(4) composite properties depended on the FDM printing temperature; filler use slightly reduced the tensile strength of bare PA-12 and larger reductions were observed for alumina. The key issue for mechanical properties was the shape and density of the CFs;
(5) along with the time of soaking in artificial saliva for both material samples: PA12-ZrO_2_ and PA12-Al_2_O_3_ decreased the Young’s modulus value, increased Elongation at UTS, while ultimate tensile strength remained at the same value. The microhardness of the samples in the initial state of PA12-ZrO_2_ was lower than that of PA12-Al_2_O_3_, while with the soaking time the hardness for PA12-Al_2_O_3_ decreased and for PA12-ZrO_2_ it increased;
(6) this study demonstrated that PA-12 can be used with CFs such as zirconia and alumina to prepare composites for biomedical applications; however, in the future, it is necessary to focus on optimizing mechanical properties via variations of filler mass proportions and 3D printing parameters. Another important issue that requires attention is an optimized 3D printing method and preparation of the base material because preparation of the FDM filament consumes large amounts of material (multi-kilogram scales), which is highly problematic for research trials.

## Figures and Tables

**Table 1:** Processing parameters of the tensile test samples.

Printing Parameters	Batch 1	Batch 2	Batch 3	Batch 4
Material	PA-ZrO_2_	PA-ZrO_2_	PA-Al_2_O_3_	PLA
Nozzle diameter (mm)	0.4 mm	0.8 mm	0.8 mm	0.8 mm
Layer thickness (mm)	0.2 mm	0.2 mm	0.2 mm	0.2 mm
Build orientation	z (vertically)	x (horizontally)	x (horizontally)	x (horizontally)
Infill density	90%	100%	100%	100%
Infill pattern	Linear (+45°/−45°)	Linear aligned (0°)	Linear aligned (0°)	Linear aligned (0°)
Outer layers	2	2	2	2
Extruder temp. (°C)	230–255 °C (5 °C increments)	250 °C	250 °C	210 °C

**Table 2:** Sample dimensions.

Dimensions of the Sample	Dimensions, mm
*l_3_*—overall length	75
*l_1_*—length of narrow parallel-sided portion	30.5
*r*—radius	37
*l_2_*—distance between broad parallel-sided portions	57.5
*b_2_*—width at ends	10
*b_1_*—width of narrow portion	5
*h*—thickness	2.35
*L_0_*—gauge length	25
*L*—initial distance between grips	54

**Table 3:** Processing parameters of the soaking test samples.

Printing Parameters		
Material	PA-ZrO_2_	PA-Al_2_O_3_
Nozzle diameter (mm)	0.5 mm	0.5 mm
Layer thickness (mm)	0.35 mm	0.35 mm
Build orientation	x (horizontally)	x (horizontally)
Infill density	100%	100%
Infill pattern	Linear aligned (0°)	Linear aligned (0°)
Outer layers	2	2
Extruder temp. (°C)	210 °C	210 °C

**Table 4:** SBF—Artificial saliva chemical composition.

Compound	Na_2_HPO_4_	NaCl	KSCN	KH_2_PO_4_	NaHCO_3_	KCl
Concentration, g/L	0.260	0.700	0.330	0.200	1.500	1.200

**Table 5:** Particle size distribution of the modified ZrO_2_ and Al_2_O_3_ powders.

Material	Dx 10 [μm]	Dx 50 [μm]	Dx 90 [μm]
ZrO_2_	2.76 (±0.01)	12 (±0)	31.86 (±0.23)
Al_2_O_3_	13.4 (±0)	21.9 (±0)	32.52 (±0.04)

**Table 6:** XPS concentration of raw and modified ceramic powders.

Sample	Concentration, %					
	Al	Si	Zr	C	N	O
Al_2_O_3__1	44.69	0.00	0.00	7.34	0.00	47.97
ZrO_2__1	0.00	0.00	30.57	22.68	0.00	46.75
Al_2_O_3__2	43.60	6.31	0.00	7.28	6.22	36.59
ZrO_2__2	0.00	7.59	23.15	24.68	7.74	36.84

**Table 7:** Hardness of tested samples.

	PA12-ZrO_2_ Initial State	PA12-ZrO_2_ 14 Days	PA12-ZrO_2_ 21 Days	PA12-ZrO_2_ 29 Days	PA12-Al_2_O_3_ Initial State	PA12-Al_2_O_3_ 14 Days	PA12-Al_2_O_3_ 21 Days	PA12-Al_2_O_3_ 29 Days
Vickers hardness, *HV_IT_*	11 ± 1	22 ± 3	9 ± 1	19 ± 2	17 ± 2	14 ± 1	5 ± 1	12 ± 1
Microhardness *H_IT_,* MPa	120 ± 8	229 ± 31	100 ± 10	202 ± 29	175 ± 20	151 ± 8	58 ± 16	128 ± 11

## Data Availability

Not applicable.
